# Rare cause of coronary artery ectasia in children: A case report of methylmalonic acidemia with hyperhomocysteinemia

**DOI:** 10.3389/fped.2022.917734

**Published:** 2022-07-22

**Authors:** Tu Juan, Chen Chao-ying, Li Hua-rong, Wan Ling

**Affiliations:** Department of Nephrology, Children’s Hospital Affiliated to Capital Institute of Pediatrics, Beijing, China

**Keywords:** methylmalonic academia, cobalamin C defect, homocystinemia, coronary artery ectasia, case report

## Abstract

**Background:**

Methylmalonic acidemia (MMA) with hyperhomocysteinemia is caused by cobalamin deficiency, mainly due to disturbance of cobalamin C (cblC) metabolism. Its clinical manifestations involve many organs. However, cases of coronary artery ectasia have been rarely reported.

**Case presentation:**

Here, we report the case of a 4-year-old girl who was hospitalized mainly because of pallor, brown urine, and fatigue, followed by hypertension, renal insufficiency, hemolytic anemia, cardiac enlargement, cardiac insufficiency, and coronary artery ectasia. Thrombotic microangiopathy (TMA) was confirmed by renal pathological examination. Metabolic examination showed hyperhomocysteinemia and methylmalonic aciduria. Furthermore, genetic assessment confirmed *MMACHC* gene variant, which confirmed the final diagnosis of a cblC defect. Intramuscular injection of hydroxy-cobalamin, oral medications of betaine, levocarnitine, folic acid, and aspirin were administered. Three months later, the patient’s condition was significantly improved. Anemia was corrected, and the renal function was normal. Heart size, cardiac function, and coronary artery structure completely returned to normal.

**Conclusion:**

The clinical manifestation of cblC deficiency is atypical. This critical condition may be associated with multiple organ involvement. A rare complication, coronary artery ectasia, can also occur. Early identification, careful evaluation, and appropriate treatment are crucially important for the improvement of this disease prognosis.

## Background

Methylmalonic acidemia (MMA) with hyperhomocysteinemia is caused by congenital abnormal metabolism of cobalamin. Cobalamin plays an important role in the metabolism of methylmalonic acid and homocysteine. Therefore, in cobalamin disorders, methylmalonic acid, and homocysteine accumulate in the body, which can be manifested as multiple organ damage (1). Notably, its clinical manifestations often lack specificity. Cardiovascular system involvement can be characterized by myocardial injury and hypertension, but coronary arteries involvement is very rare (2). Here, we report the case of a 4-year-old girl with MMA complicated with hyperhomocysteinemia, caused by a cblC congenital defect. To the best of our knowledge, this is the first report of MMA combined with coronary artery ectasia.

## Patient information

A 4-year-old girl was admitted to hospital because of pale complexion and brown urine, accompanied by abdominal pain, vomiting, and fatigue. She was the second child of non-consanguineous parents whose first daughter was healthy. Her perinatal medical and family histories were unremarkable. She had normal level of mental and motor development for her age. Three months earlier, the child had undergone hemoglobin tests in local hospital, with a result of 84 g/L [mean corpuscular volume (MCV) 101 fL (reference value range 80–100 fL), mean corpuscular hemoglobin concentration (MCHC) 307 g/L (reference value range 310–350 g/L), serum iron 7.2 μmol/L (reference value range9–27 μmol/L)]. In addition, considering her poor appetite she was diagnosed with “iron deficiency anemia” and iron supplement treatment was administered. One month later, the anemia of the girl was improved with the hemoglobin level of 113 g/L (MCV103 fL, MCHC318 g/L).

Physical examination revealed normal body temperature, pulse 122 times/min, blood pressure 140/94 mmHg, Height 100 cm, Weight 14.2 kg, BMI 14.2 kg/m^2^, BMI-for-age z score −1.1. The girl had pale appearance, with swelling in face and lower extremities. The first heart sound (S1) was low and dull. No murmurs, gallops, or rubs were detected.

Laboratory examinations showed that the patient had hypocomplementemia C3 0.73 g/L (normal rage 0.9–1.8 g/L). Autoantibodies like antinuclear antibody, anti-DNA, anti-neutrophil cytoplasmic antibody and anti-glomerular basement membrane antibody were negative. Pathogenic tests including hepatitis B virus, hepatitis C virus, cytomegalovirus, Epstein-Barr virus and parvovirus B19 were also negative. And blood gas analysis was normal.

## Hematological assessment

The complete blood count showed macrocytic anemia. The hemoglobin level was 62 g/L. The MCV was 107 fL (reference value range 80–100 fL), MCHC was 369 g/L (reference value range 310–350 g/L), and the reticulocytes were increased to 9.6%. The platelet count was 194*10^9^/L, and the white blood cell count is normal. Red blood cell fragments were visible in the peripheral blood smears. Serum lactate dehydrogenase (LDH) was significantly increased to 939 U/L (normal value range 80–300 U/L). The levels of the serum vitamin B12 [19 ng/mL (normal value range 3.1–19.9 ng/mL)], folic acid [700 pg/ml (normal value range 197–775 pg/mL)], ferritin [114 ng/mL (normal value range 10–120 ng/mL)], and transferrin saturation [23% (normal value range 20–55%)] were normal. Bone marrow examination revealed significant erythroid hyperplasia. Large red blood cells were also observed, along with a normal number of megakaryocytes and platelets. The Coomb’s test was negative and the coagulation index was normal. Combined with the aforementioned findings of the blood system examinations, we concluded that the child had non-immune hemolytic anemia.

## Kidney assessment

**FIGURE 1:**
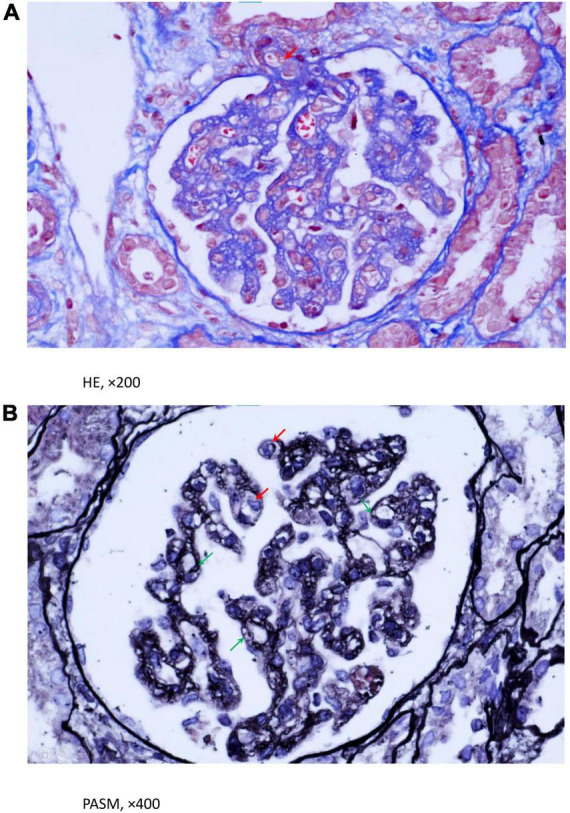
**(A,B)** Light microscopy evaluation of renal biopsy using HE and PASM staining. A. HE staining implied that some lumen occlusion and intima edema was in partial afferent arteriole (Red arrowhead). **(B)** PASM staining showed endotheliocytes were swollen and proliferative, partial capillary loops were compressed and narrowed (Red arrowhead), GBM were incrassated and degenerated, with lamina rata internal expansion and formation of double contours (Green arrowhead). **(A)** (HE, ×200), **(B)** (PASM, ×400).

The results of the kidney examinations were as follows: serum creatinine 124 μmol/L (reference value range 19–44 μmol/L), urinary total protein excretion reached 1,632 mg/d, urine centrifugal microscopy showed red blood cell (RBC) 40–50/high-power field (hpf). Additionally, the child had non-immune hemolysis with abnormal renal function, so hemolytic uremic syndrome (HUS) was highly suspected, although she was not accompanied by the typical manifestation of HUS—thrombocytopenia. Without any contraindication, renal biopsy was performed on day 8 after the girl’s admission. Light microscopy showed proliferated and swelled endothelial cells, some capillary loops were compressed and narrowed, the lumen of some of the afferent arterioles was occluded, and the intima was edematous, which were consistent with thrombotic microangiopathy (Figures 1A,B). We determined the activity of the complement factor H, the serum level of antibody against factor H and C5b-9, and all showed normal. Normal ADAMTS-13 activity and negative ADAMTS-13 antibody ruled out thrombotic thrombocytopenic purpura.

## Cardiac assessment

N-terminal pro-brain natriuretic peptide (NT-BNP) 1,290 pg/mL (reference value range < 289 pg/mL), high-sensitivity troponin T (hsTnT)17 ng/L (reference value range ≤ 14 ng/L). The echocardiography findings showed that the left atrium and left ventricle were enlarged. The ventricular wall motion amplitude was reduced, and the left ventricular systolic function was decreased [ejection fraction, (EF) 44%]. Vascular ultrasound revealed: left main coronary artery [diameter 3.6 mm (Z = + 3.59)], anterior descending branch [diameter 2.7 mm (Z = + 2.57)], and circumflex artery [diameter 2.5 mm (*Z* = + 2.42)] dilation. The walls of these blood vessels were not smooth.

## Metabolism testing and diagnostic assessment

**FIGURE 2:**
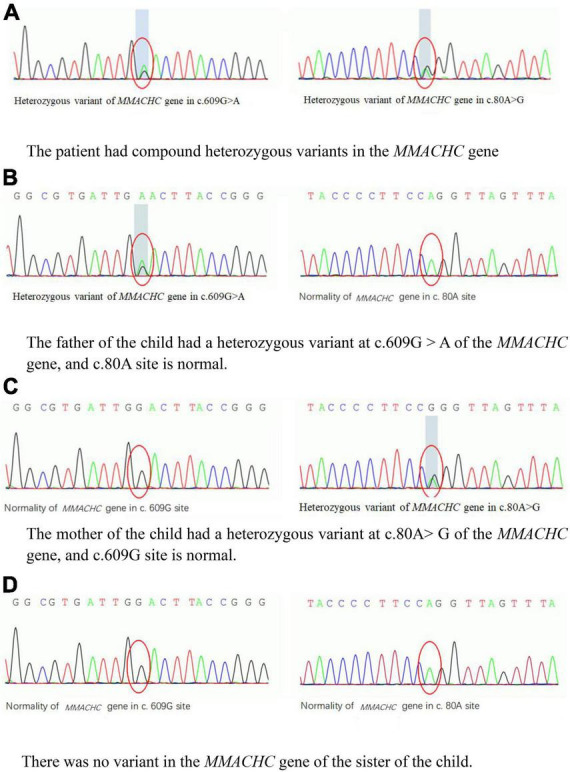
**(A–D)** The variants in the *MMACHC* gene of the patient and her family. HGB, hemoglobin; SCr, serum creatinine; SHcy, serum homocysteine. **(A)** The patient had compound heterozygous variants in the *MMACHC* gene. **(B)** The father of the child had a heterozygous variant at c.609G > A of the *MMACHC* gene, and c.80A site is normal. **(C)** The mother of the child had a heterozygous variant at c.80A > G of the *MMACHC* gene, and c.609G site is normal. **(D)** There was no variant in the *MMACHC* gene of the sister of the child.

The specific biochemical examination revealed that the level of serum homocysteine was significantly increased up to 216.7 μmol/L (normal range 3.7–13.9 μmol/L). The level of propionyl carnitine (C3) in the blood reached 7.31 μmol/L (normal range 0–5.41 μmol/L); the ratio C3/acetyl carnitine (C2) was 0.31 (normal range 0.03–0.20). The concentration of urinary methylmalonic acid reached 43.05 (normal range 0–5.34). Using next-generations sequencing, we detected compound heterozygous variants in the *MMACHC* gene. The variant sites c.80A > G (p.Q27R) and c.609G > A (W203X) were derived from the father and mother, respectively (Figures 2A–D). According to the American College of Medical Genetics (ACMG) guideline, they all were pathogenic variants (3, 4). In addition, we did not find any pathogenic variant in genes of atypical hemolytic uremic syndrome (aHUS). Until then, the diagnosis of the child was clear: methylmalonic acidemia with homocysteinemia (cblC type) and secondary TMA.

## Therapy and follow-up examinations

The child was given active treatment to correct the metabolic disorder of cblC defect. The specific treatments included intramuscular injection of hydroxy-cobalamin (1 mg/day), oral betaine (300 mg/kg/day), carnitine (75 mg/kg/day), and folic acid (5 mg/d); ramipril and amlodipine were applied to lower the high blood pressure. Considering that the child had coronary artery ectasia, a low-dose aspirin (3–5 mg/kg/day) was also used as an antiplatelet aggregation agent.

**FIGURE 3:**
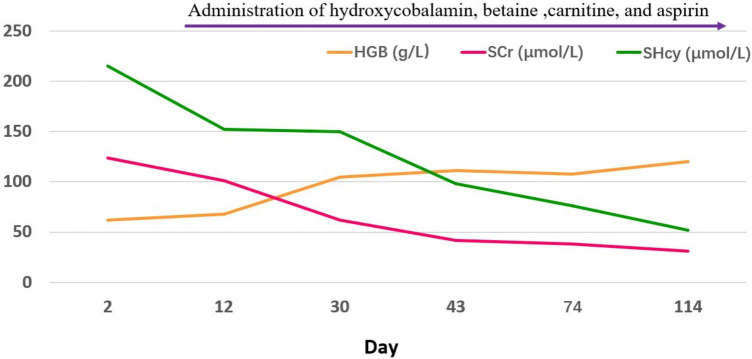
The changes of hemoglobin, serum creatinine, and serum homocysteine.

One month after the start of the above treatment, the child’s heart and kidney functions were significantly improved, and hemolytic anemia was corrected. Three months later, the child visited the outpatient clinic for a follow-up examination. She was lively and talkative, with normal blood pressure, negative urine protein (Figure 3 and Table 1). Her blood homocysteine level decreased, and the condition of her coronary artery dramatically returned to normal: left main coronary artery diameter 2.6 mm (Z = + 1.20), anterior descending branch diameter 2.0 mm (Z = + 1.80)], circumflex artery diameter 1.5 mm (Z = −0.28)].

**TABLE 1:** The changes of clinical and lab value before and after treatment.

Clinical and lab value	Baseline value	One month after treatment	Three months after treatment
HGB (g/L)	62	102	118
Bp (mmHg)	140/94	125/85	102/62
EF (%)	44	48	56
Egfr (ml/min/1.73 M^2^)	29.4	57.0	89.6
UPr/Cr (mg/mg)	9.07	1.4	0.2

HGB, hemoglobin; Bp, blood pressure; EF, ejection fraction; eGFR, estimated glomerular filtration rate; UPr/Cr, urinary protein creatinine ratio.

## Discussion and conclusion

CblC deficiency is an autosomal recessive hereditary disease, which is caused mainly by variants in the MMACHC gene. The protein it encodes is located in the cytoplasm and plays an important role in the transport of cobalamin within the cytoplasm as well as into the mitochondria, thereby affecting mitochondrial cobalamin metabolism also. MMACHC disorders result in decreased intracellular production of adenosylcobalamin and methylcobalamin (MeCbl), which ultimately affect the metabolism of methylmalonic acid and homocysteine (1, 2). The incidence of cblC deficiency is estimated to be within the range 1/60,000–36,000. It is the most frequent type of methylmalonic acidemia in China (5). Due to the accumulation of hyperhomocysteinemia, methylmalonic acid and secondary metabolites in the body, patients experience multiple organ involvement (1).

The patient had a late onset age of 4 years old. The first symptoms are pale skin, brown urine, and digestive tract involvement, and then the manifestation of TMA was presented. TMA is an important complication of cblC deficiency. The vascular damage caused by homocysteinemia plays an important role in its pathogenesis. An earlier review of published case reports and case series established that 36 patients with cblC deficiency had TMA, which was manifested with hemolytic anemia, thrombocytopenia, and peripheral red blood cell fragments (6). Our case had the clinical features of TMA, but without thrombocytopenia, and the diagnosis was finally confirmed by renal biopsy. Thrombocytopenia is a typical clinical manifestation of TMA, but many reports have shown that there is no thrombocytopenia in cblC deficiency combined with TMA (7, 8). Platelet aggregation and consumption have been speculated not to be the only mechanism of microangiopathy in such patients. Endothelial cell swelling and smooth muscle cell proliferation also play important roles. Anemia was another important clinical feature of this girl, manifested as macrocytic anemia. Hematological manifestations are very common in children with cblC deficiency, and it accounted for 83.3% of the cases described in a previous report from China (5). Apart from hemoclasis generated by TMA, the causes of anemia are also related with obstruction of DNA synthesis due to cobalamin disorders, eventually impairing cell division and inducing megaloblastic anemia.

In addition, the cardiovascular involvement in this case was very obvious. Cardiac complications of cblC deficiency often manifest as congenital heart disease, dilated cardiomyopathy, pulmonary hypertension, and arterial hypertension (2). To the best of our knowledge, this is the first report of MMA combined with coronary artery ectasia. Coronary artery ectasia in children is always secondary to Kawasaki disease, and few of cases are also secondary to other vasculitis types such as systemic lupus erythematosus and polyarteritis nodosa, as well as to infections such as Epstein-Barr virus and syphilis (9, 10). Dilated coronary arteries can form local thrombus, vascular stenosis, and myocardial ischemia, leading to poor prognosis (11). Geraghty et al. reported a case of coronary artery disease in a child with cblC deficiency, who also had TMA, nervous system damage, and eventually died. The autopsy revealed intimal hyperplasia of the right coronary artery and local fibrosis of the endocardium (12). The pathogenesis of coronary artery ectasia may be related to hyperhomocysteinemia, which plays an important role in the occurrence and development of vascular lesions through cytotoxicity, promoting thrombosis and atherosclerosis (13).

The genetic test of the child indicated that the MMACHC gene has compound heterozygous variant s. The c.609 G > A is the most common variant site in Chinese patients with cblC deficiency, accounting for 48–55.4% of all cases. Patients with this variant often manifest as early onset methylmalonic acidemia. The c.80A > G variant is also common, with an incidence of cblC defects in the Chinese population within the range 5.1–9.09% (4, 5, 14). Previous reports have described the cases of patients with *MMACHC* gene variants, similarly to this child, which showed compound heterozygous variants of c.80A > G (p.Q27R) and c.609G > A (W203X). The disorder in such patients had often been accompanied by HUS or pulmonary hypertension. Therefore, it has been speculated that this type of compound heterozygous variant sites has susceptibility to microvascular damage (6, 15, 16).

In summary, we report a case of a 4-year-old girl diagnosed with cblC disorder, with clinical manifestations of critical illness, including renal insufficiency, cardiac insufficiency, hemolytic anemia, and coronary artery dilation. Despite the serious condition of this child at the onset of the illness, a good prognosis was finally achieved by effective treatment. Therefore, in children with multiple damage, especially with TMA, it is critical to consider cblC deficiency. Metabolic analysis and gene detection are essential for making the diagnosis. In addition, it is necessary to conduct a comprehensive cardiac assessment in such patients. Furthermore, coronary B-ultrasound examination findings are important for the provision of timely treatment and an appropriate follow-up duration.

## Data availability statement

The datasets for this article are not publicly available due to concerns regarding participant/patient anonymity. Requests to access the datasets should be directed to the corresponding author.

## Ethics statement

The studies involving human participants were reviewed and approved by the Institutional Ethics Committee of the Capital Institute of Pediatrics. Written informed consent to participate in this study was provided by the participants’ legal guardian/next of kin. Written informed consent was obtained from the individual(s), and minor(s)’ legal guardian/next of kin, for the publication of any potentially identifiable images or data included in this article.

## Author contributions

TJ drafted the initial manuscript. CC-Y reviewed and revised the manuscript. LH-R and WL collected data of the patient. All authors approved the final manuscript as submitted and agreed to be accountable for all aspects of the work.

## Conflict of interest

The authors declare that the research was conducted in the absence of any commercial or financial relationships that could be construed as a potential conflict of interest.

## Publisher’s note

All claims expressed in this article are solely those of the authors and do not necessarily represent those of their affiliated organizations, or those of the publisher, the editors and the reviewers. Any product that may be evaluated in this article, or claim that may be made by its manufacturer, is not guaranteed or endorsed by the publisher.
